# Supplementary material for: Assessing the impact of timely diagnosis on psychological outcomes and quality of life for cancer patients: A scoping review
Source: PLoS One. 2026 Mar 16;21(3):e0338136. doi: 10.1371/journal.pone.0338136 (PMC12991267; doi:10.1371/journal.pone.0338136)
Supplement: S1 Table — (DOCX) [file pone.0338136.s001.docx]

**Supplement S1. Search Strategy**

The following electronic databases were searched: MEDLINE, Cumulative Index for Nursing and Allied Health Professionals (CINAHL), and PsychInfo via EBSCOhost. The most recent search was conducted in November 2024.

MEDLINE and CINAHL were searched together, followed by a second search using only PsycInfo. The search strategy for MEDLINE and CINAHL is as follows:

**1** cancer OR neoplasm OR tumo* OR carcinoma OR malignancy

AND

**2** "early diagnos*" OR "delay*diagnos*” OR “late diagnos*” OR “time to diagnos*” OR “time to consult*” OR “time to treat*” OR “time to refer*” OR “time to present*” OR “early referral” OR “earl* consultation*” OR “delay* consultation*” OR “late consultation*” OR “time interval*” OR consult* OR "diagnos* delay" OR “new* diagnos*”

AND

**3** psycholog* OR emot* OR “mental health” OR “well being” OR coping OR resilience OR adjustment* OR adaptation* OR “quality of life” OR “patient experience*”
