# Supplementary material for: Assessing the impact of timely diagnosis on psychological outcomes and quality of life for cancer patients: A scoping review
Source: PLoS One. 2026 Mar 16;21(3):e0338136. doi: 10.1371/journal.pone.0338136 (PMC12991267; doi:10.1371/journal.pone.0338136)
Supplement: S2 Table — (DOCX) [file pone.0338136.s002.docx]

**Supplement S2. Inclusion and Exclusion Criteria**

| **Inclusion Criteria** |
| --- |
| Articles that explore, measure, or focus on the time to diagnosis of cancer, psychological outcomes (see Table 1 for various definitions) in relation to the diagnosis of cancer, and the links between them. |
| Written in English |
| Published in academic journals |
| Published from 2007 onwards |
| Relates to the diagnostic *process* or the *perceived* process |

| **Exclusion Criteria** |
| --- |
| Grey literature |
| No psychological component |
| Wrong subject matter (e.g., validation of a tool/ focus on medication) |
| Relating to severe mental illness |
| Does not focus on the target population (children, parents, caregivers etc.) |
| Relating to psychological variables pre-cancer diagnosis |
| Relating to illnesses other than cancer |
| The wrong publication type (e.g., protocols, reviews) |
| Only presents the stage of cancer associated with the diagnosis |
