# Supplementary material for: Assessing the impact of timely diagnosis on psychological outcomes and quality of life for cancer patients: A scoping review
Source: PLoS One. 2026 Mar 16;21(3):e0338136. doi: 10.1371/journal.pone.0338136 (PMC12991267; doi:10.1371/journal.pone.0338136)
Supplement: S3 Table — (DOCX) [file pone.0338136.s003.docx]

**Supplement S3. Criteria for assessing ‘good studies’ developed by Neal et al. (16)**
