# Supplementary material for: Assessing the impact of timely diagnosis on psychological outcomes and quality of life for cancer patients: A scoping review
Source: PLoS One. 2026 Mar 16;21(3):e0338136. doi: 10.1371/journal.pone.0338136 (PMC12991267; doi:10.1371/journal.pone.0338136)
Supplement: S4 Table — (DOCX) [file pone.0338136.s004.docx]

**Supplement S4. Quality Appraisal using the MIXED METHODS APPRAISAL TOOL (MMAT) VERSION 2018, Hong et al. (45)**

(L=Lehto et al., M1=Malmstrom et al., M2=Mendonca et al., M3=Miles et al., R=Robinson et al., S=Soomers et al.)

| **Category of study designs** | **Methodological quality criteria** | **Responses** | | | |
| --- | --- | --- | --- | --- | --- |
|  |  | Yes | No | Can’t tell | Comments |
| Screening questions  (for all types) | S1. Are there clear research questions? | L,  M1, M2, M3,  R, S |  |  | Lehto et al. had an exploratory aim rather than specific questions |
|  | S2. Do the collected data allow to address the research questions? | L,  M1, M2,  M3, R, S |  |  |  |
|  | *Further appraisal may not be feasible or appropriate when the answer is ‘No’ or ‘Can’t tell’ to one or both screening questions.* | | | | |
| 1. Qualitative | 1.1. Is the qualitative approach appropriate to answer the research question? | M1 |  |  |  |
|  | 1.2. Are the qualitative data collection methods adequate to address the research question? | M1 |  |  |  |
|  | 1.3. Are the findings adequately derived from the data? | M1 |  |  |  |
|  | 1.4. Is the interpretation of results sufficiently substantiated by data? | M1 |  |  |  |
|  | 1.5. Is there coherence between qualitative data sources, collection, analysis and interpretation? | M1 |  |  |  |
| 2. Quantitative randomized controlled trials | 2.1. Is randomization appropriately performed? |  |  |  |  |
|  | 2.2. Are the groups comparable at baseline? |  |  |  |  |
|  | 2.3. Are there complete outcome data? |  |  |  |  |
|  | 2.4. Are outcome assessors blinded to the intervention provided? |  |  |  |  |
|  | 2.5 Did the participants adhere to the assigned intervention? |  |  |  |  |
| 3. Quantitative non-randomized | 3.1. Are the participants representative of the target population? |  |  |  |  |
|  | 3.2. Are measurements appropriate regarding both the outcome and intervention (or exposure)? |  |  |  |  |
|  | 3.3. Are there complete outcome data? |  |  |  |  |
|  | 3.4. Are the confounders accounted for in the design and analysis? |  |  |  |  |
|  | 3.5. During the study period, is the intervention administered (or exposure occurred) as intended? |  |  |  |  |
| 4. Quantitative descriptive | 4.1. Is the sampling strategy relevant to address the research question? | M2, M3,  R, S |  |  |  |
|  | 4.2. Is the sample representative of the target population? | M2, M3, R, S |  |  |  |
|  | 4.3. Are the measurements appropriate? | M2, M3, R, S |  |  |  |
|  | 4.4. Is the risk of nonresponse bias low? | M2, M3, R, S |  |  |  |
|  | 4.5. Is the statistical analysis appropriate to answer the research question? | M2, M3, R, S |  |  |  |
| 5. Mixed methods | 5.1. Is there an adequate rationale for using a mixed methods design to address the research question? | L |  |  |  |
|  | 5.2. Are the different components of the study effectively integrated to answer the research question? | L |  |  |  |
|  | 5.3. Are the outputs of the integration of qualitative and quantitative components adequately interpreted? | L |  |  |  |
|  | 5.4. Are divergences and inconsistencies between quantitative and qualitative results adequately addressed? | L |  |  |  |
|  | 5.5. Do the different components of the study adhere to the quality criteria of each tradition of the methods involved? | L |  |  |  |

| **Paper** | **Does the study report time intervals in compliance with the definitions in the Aarhus statement?** (52) | **Does the study report dates (bodily change/presentation/diagnosis/referral)?** | **Did the study use appropriate data collection methods for the time intervals?** |
| --- | --- | --- | --- |
| Lehto | No. Unreported | No. Unreported | No. They did not measure time intervals |
| Malmstrom | No. Unreported | No. Patient perceived length | No. They did not measure time intervals |
| Mendonca | Yes. (Primary care) | No. Unreported | Yes. Quantitative definition (number of pre-referral GP appointments) |
| Miles | No. Unreported | No. Patient perceived length | No. They did not measure time intervals |
| Robinson | Yes. (Total delay) | No. Unreported | Yes (number of weeks between first cancer symptom and initiation of treatment) |
| Soomers | Yes. (Patient and diagnostic) | No. Patient perceived length | Yes. ‘*Intervals were patient-reported, and the answers were categorical. The meaning of each interval was explained in detail to the patients to overcome interpretation issues’ (49)* |
