# Supplementary material for: Assessing the impact of timely diagnosis on psychological outcomes and quality of life for cancer patients: A scoping review
Source: PLoS One. 2026 Mar 16;21(3):e0338136. doi: 10.1371/journal.pone.0338136 (PMC12991267; doi:10.1371/journal.pone.0338136)
Supplement: S6 Table — (DOCX) [file pone.0338136.s006.docx]

|  | **Lehto et al.** | **Malmstrom et al.** | **Mendonca et al.** | **Miles et al.** | **Robinson et al.** | **Soomers et al.** |
| --- | --- | --- | --- | --- | --- | --- |
| **Study conceptualisation of timely diagnosis** | Patient reported | Patient reported | A quantitative definition used as a proxy | Patient reported | Time intervals | Time intervals |
| **Specific definition of timely diagnosis** | Only one participant quote: *"did it get diagnosed early enough"?* | Answers to the interview question: *‘Could you start by telling me, in your own words and in as much detail as you want, about everything that has happened since you first started to suspect there might be a problem with your health?’* | ‘three or more pre-referral consultations with a GP’. | Answers to the survey question, *‘Do you think your cancer could have been diagnosed sooner than it was?’* | The number of weeks between the first cancer symptoms and the initiation of treatment *(taken from electronic health records)* | The time from the first symptom until the first presentation to a doctor (patient interval), and the time from this first presentation until pathologic diagnosis (diagnostic interval) *(taken from electronic health records)* |

Supplement S6. Conceptualisation of timely diagnosis in studies which met our inclusion criteria
