# Supplementary material for: Assessing the impact of timely diagnosis on psychological outcomes and quality of life for cancer patients: A scoping review
Source: PLoS One. 2026 Mar 16;21(3):e0338136. doi: 10.1371/journal.pone.0338136 (PMC12991267; doi:10.1371/journal.pone.0338136)
Supplement: Attachment — Submitted filename: Response to Reviewers_.docx [file pone.0338136.s009.docx]

| **Feedback** | **Author Response** |
| --- | --- |
| Methods: Mention the specific date when the last literature search was conducted. | Line 160 has now been updated to specify the last literature search date of the 25^th^ of November. |
| Methods: Description of the identification of records by snowballing from previous studies should be provided in the main text. This is illustrated in the PRISMA flowchart. | Line 205 of the manuscript, and the PRISMA flowchart have now been updated for clarity. |
| PRISMA flowchart: Mention the specific number of records identified at MEDLINE, CINAHL, and PsycINFO (First box on the left-hand corner of the screen). | The PRISMA flowchart has now been updated to illustrate that MEDLINE and CINAHL (searched together) retrieved 2850 articles, and PsycINFO retrieved 2547. |
| PRISMA flowchart: Cross-check all the numbers provided in this Figure for accuracy. | Thank you for highlighting this. The number of reports excluded has been updated. |
| Supplementary File 1/Methods: Was the same string of search terms employed in all 3 databases? Related information must be provided in the main text. Besides, the information provided in the Supplementary File 1 lacks clarity. | Thank you for this. Both the text from line 159 and the supplementary file have now been updated to clarify the process. |
| Page 20, Line 171, S2_In.Exclusion_Criteria: Indicate “Inclusion & Exclusion Criteria”. Similar corrections should be made elsewhere. | All instances of using this phrase (including the supplementary materials) have now been updated to ‘Inclusion & Exclusion Criteria’. |
| Supplementary File 2: The presentation of the inclusion and exclusion criteria should be changed. Do not place them side by side in 2 columns. Place the list of exclusion criteria below the list of inclusion criteria, clearly differentiating between the two. | This table has now been split into two separate tables, with the exclusion criteria below the inclusion criteria, as requested. |
| Page 39: Provide the complete legend/caption/title for the Supplementary Files. | All supplementary articles have been modified. |
| Supplementary File 5: The footnotes provided should correspond to the information provided in the ‘Checklist’. For example, there seems to be no mention of JBI in the ‘Checklist’. | This was an oversight and has been updated. Thank you for highlighting this. |
| Please Check the following references - reference numbers - 6, 7, 8, 9, 13, 14, 15, 18, 36, 39, 43, 45, 50, 62. PMID not uniformly reported. Recommended to follow uniform referncing style. | References should all now be correct and conform to PLOS One’s referencing style. |

**Response to Reviewers**
