# Supplementary material for: Assessing the impact of timely diagnosis on psychological outcomes and quality of life for cancer patients: A scoping review
Source: PLoS One. 2026 Mar 16;21(3):e0338136. doi: 10.1371/journal.pone.0338136 (PMC12991267; doi:10.1371/journal.pone.0338136)
Supplement: Attachment — Submitted filename: Response to Reviewers.docx [file pone.0338136.s010.docx]

| **Journal Requirements:** | **Author Response** |
| --- | --- |
| 1. If the reviewer comments include a recommendation to cite specific previously published works, please review and evaluate these publications to determine whether they are relevant and should be cited. There is no requirement to cite these works unless the editor has indicated otherwise. | No additional references were added in response to the reviewers’ comments. |
| 2. Please review your reference list to ensure that it is complete and correct. If you have cited papers that have been retracted, please include the rationale for doing so in the manuscript text, or remove these references and replace them with relevant current references. Any changes to the reference list should be mentioned in the rebuttal letter that accompanies your revised manuscript. If you need to cite a retracted article, indicate the article’s retracted status in the References list and also include a citation and full reference for the retraction notice. | We have reviewed the reference list for completeness and accuracy. |
| **Additional Academic Editor Comments:** | **Author Response** |
| The major issue remains the lack or inconsistency of the definition or explanation for "timely diagnosis" of cancer. The related information put up in Table 1 is confusing and lacks clarity (specifically, the use of phrases like "time since diagnosis" and "ED" in the first row). Make sure to rearrange the order of columns. Besides, club information related to "authors, year of publication, country" in one column, and information on "sample, cancer type, study design, analysis" (follow the same sequence for all the references) in another column. | Thank you - this is an excellent suggestion, and we have reorganised Table 1, beginning on p10, according to these suggestions. We have also decided to simplify and create a single column for the definition of timely diagnosis, providing additional narrative in the text of the results. |
| The answers to the question "How have studies conceptualised timely diagnosis?" must be detailed in the text ('results' section). Provide more related information. | We have now included more related information on how studies conceptualised timely diagnosis in the text of the results section on p16. |
| Include a separate new Table summarizing the findings related to conceptualization of timely diagnosis in the studies that met the inclusion criteria. | We have now provided a Table summarising the main findings related to the conceptualisation of timely diagnosis (see S6_Conceptualisation_of_Timely_Diagnosis). |
| The related limitations must be further elaborated in the 'limitations' section. | The discussion of limitations related to conceptualising and measuring timely diagnosis is on p 20 and 21 and see also below. |
| In relation to the definition/explanation of/for "timely diagnosis" of cancer, what are the recommendations suggested (by the authors of the present review) to be followed in future studies? | Following on from above, we have added an overall summary to provide a greater steer for future studies, beginning on p20. |
